# Comparison of radial peripapillary capillary density results of individuals with and without *Helicobacter pylori* infection

**DOI:** 10.5935/0004-2749.202200106

**Published:** 2022

**Authors:** Cemile Ucgul Atilgan, Ahmet Yozgat, Pinar Kosekahya, Yasin Sakir Goker, Emine Sen, Esat Yetkin, Benan Kasapoglu

**Affiliations:** 1 Ulucanlar Eye Training and Research Hospital, Altindag, Ankara, Turkey; 2 Department of Gastroenterology, Oncology Training and Research Hospital, Ankara, Turkey

**Keywords:** Glaucoma, *Helicobacter pylori*, Tomography, optical coherence, Capillary density, Retinal nerve fiber layer thickness, Optic nerve/pathology, Nerve fiber/pathology, Glaucoma, *Helicobacter pylori*, Tomografia de coerência óptica, Densidade capilar, Espessura da camada de fibras nervosas da retina, Nervo óptico/patologia, Fibras nervosas/ patologia

## Abstract

**Purpose:**

To evaluate the radial peripapillary capillary density using optical coherence tomography angiography in patients with and without *Helicobacter pylori* infection.

**Methods:**

This prospective, cross-sectional study comprised 52 patients (52 eyes: Group 1) and 38 patients (38 eyes: Group 2) with and without *H. pylori* infections, respectively. The radial peripapillary capillary density and retinal nerve fiber layer thickness in 4 equal quadrants and 2 equal hemispheres in the peripapillary region were calculated using optical coherence tomography angiography. The optic nerve head parameters of the patients were also assessed.

**Results:**

The groups were similar in terms of age, gender, and the optic nerve head parameters. The radial peripapillary capillary densities in the superior hemisphere and quadrant were significantly lower in Group 1 than in Group 2 (p=0.039 and p=0.028, respectively) and were positively correlated with the superior hemisphere’s retinal nerve fiber layer thickness (p<0.001 and p<0.001, respectively). Similarly, the radial peripapillary capillary densities in the inferior hemisphere and quadrant were also significantly lower in Group 1 compared to Group 2 (p=0.03 and p=0.017, respectively) and were positively correlated with the inferior hemisphere’s retinal nerve fiber layer thickness (p<0.001 and p<0.001, respectively). The retinal nerve fiber layer thickness in the nasal and temporal quadrants were significantly decreased in Group 1 when compared to Group 2 (p=0.013 and p=0.022) and were positively correlated with the corresponding radial peripapillary capillary densities of the 2 quadrants (p=0.002 and p=0.022).

**Conclusion:**

The decreased radial peripapillary capillary density in the *H. pylori*-positive patients suggests that *H. pylori* may play a role in the etiopathogenesis of glaucoma.

## Abstract (pt)

**Objetivos:**

Avaliar a densidade capilar peripapilar radial de pacientes com e sem infecção por *Helicobacter pylori (H. pylori)* por meio de angiotomografia de coerência óptica.

**Métodos:**

Cinquenta e dois olhos de 52 pacientes com infecção por *H. pylori* (Grupo 1) e 38 olhos de 38 pacientes sem infecções por *H. pylori* (Grupo 2) foram incluídos neste estudo prospectivo e transversal. A densidade capilar peripapilar radial (%) e a espessura da camada de fibra nervosa retiniana (µm) em 4 setores iguais e 2 hemisférios iguais foram calculados automaticamente na região peripapilar por angiotomografia de coerência óptica. Os parâmetros da cabeça do nervo óptico dos pacientes também foram avaliados.

**Resultados:**

Os grupos foram semelhantes em relação aos parâmetros: idade, sexo e cabeça do nervo óptico. As densidades capilares peripapilares radiais no hemisfério superior, hemisfério inferior, quadrante superior e quadrante inferior foram significativamente menores no Grupo 1 do que no Grupo 2 (p=0,039, p=0,03, p=0,028 e p=0,017 respectivamente). As densidades capilares peripapilares radiais, tanto no hemisfério superior quanto no quadrante superior, foram correlacionadas positivamente com a espessura da camada de fibra nervosa da retina do hemisfério superior (p<0,001 e p<0,001). As densidades capilares peripapilares radiais no hemisfério inferior e no quadrante inferior foram positivamente correlacionadas com a espessura da camada do nervo retiniano do hemisfério inferior (p<0,001 e p<0,001). A espessura da camada da fibra nervosa retiniana nos quadrantes nasal e temporal diminuiu significativamente no Grupo 1 quando comparado ao Grupo 2 (p=0,013 e p=0,022), e esses valores foram positivamente correlacionados com as densidades capilares peripapilares radiais correspondentes nos quadrantes nasal e temporal (p=0,002 e p=0,022).

**Conclusão:**

A diminuição das densidades capilares peripapilares radiais nos olhos de indivíduos positivos para *H. pylori* sugere que *H. pylori* pode desempenhar um papel na etiopatogenia do glaucoma.

## INTRODUCTION

*Helicobacter pylori* (H. pylori), a spiral-shaped, gramne gative bacterium living in the stomach mucosa, can cause various gastrointestinal disorders such as chronic gastritis, peptic ulcer disease, and many gastrointestinal malignancies. In addition to these common illnesses, extra-gastrointestinal manifestations of *H. pylori* have recently drawn the interest of many researchers^(1)^. A positive association of *H. pylori* with some eye diseases like glaucoma, central serous chorioretinopathy, blepharitis, and uveitis has been emphasized previously^(2-6)^.

Glaucoma is a progressive optic neuropathy that insidiously causes severe visual impairment. Although an increase in the intraocular pressure (IOP) is the most important risk factor for glaucoma, impaired microcirculation of the optic nerve head (ONH), autoimmune mechanisms, excitotoxicity, and oxidative stress may all cause glaucoma^(7-11)^. It has been recently claimed that an *H. pylori* infection causes primary open-angle glaucoma (POAG), normal-tension glaucoma (NTG), and pseudoexfoliative glaucoma (PxG)^(12-14)^. Although studies related to the mechanisms underlying the pathogenesis of glaucoma by *H. pylori* are controversial, the vasoactive and inflammatory mediators secreted by *H. pylori* are thought to cause glaucomatous optic neuropathy by inducing apoptosis^(15)^. However, long-term *H. pylori* infections can directly or indirectly cause endothelial dysfunction resulting in occlusive arterial diseases, such as coronary artery disease and atherosclerosis^(16)^.

The radial peripapillary capillary (RPC) layer is the most superficial layer extending between the inner limiting membrane and the outer border of the retinal nerve fiber layer (RNFL), feeding the RNFL surrounding the ONH. Decreased RPC densities (RPCDs) in patients with glaucoma and glaucomatous optic neuropathy have been reported recently^(17-19)^. Optical coherence tomography angiography (OCTA) is a novel non-invasive imaging technique that allows retinal and choroidal angiography, which can be used for visualization and quantification of the RPC layer and identify defects^(20)^.

In this study, we compared the peripapillary microcirculation between *H. pylori*-positive and *H. pylori* negative patients without glaucoma, using OCTA. The aim was to assess whether *H. pylori* induced glaucoma by reducing the RPCD.

## METHODS

This prospective, cross-sectional study was conducted at a tertiary care hospital in accordance with the Declaration of Helsinki guidelines. The local ethics committee’s approval for the conduct of the study and informed consent from each participant were obtained before study initiation.

This study consisted of 90 patients (90 eyes) who were divided into two groups: 52 patients who tested positive for *H. pylori* infections (Group 1) and 38 control patients who did not have *H. pylori* infections (Group 2). The exclusion criteria were as follows: patient’s aged <18 or *>*60 years; history of smoking, drug and/ or alcohol addiction, and systemic disorders such as diabetes mellitus, hypertension, and cardiovascular diseases; history of intraocular surgery; presence of media opacities such as cataract or band keratopathy that rendered funduscopic examination or OCTA of the ONH impossible, and any vitreoretinal disorders; diagnosed with glaucoma and/or a glaucomatous ONH (cup-todisc [C/D] ratio *>*0.6, vertical cup asymmetry *>*0.2, and neuroretinal rim loss or notching); and refractive error above -2/+2 diopters.

*H. pylori* infection was confirmed via histological examination of tissue samples obtained from gastroscopy. After intravenous administration of midazolam (Dormicum, Roche, Switz) at a dosage of 3-5 mg based on the patient’s weight, the nasopharynx was anesthetized with xylocaine spray, and gastroscopy was performed. Four biopsy specimens were obtained from the antrum and body of the stomach^(21)^. Hematoxylin and eosin (H&E) staining was used for the histological examination. Immunohistochemical tests were performed when the H&E staining was insufficient for detecting the bacteria.

All the patients underwent complete ophthalmological evaluation including the best-corrected visual acuity (BCVA) using the Snellen chart, slit-lamp examination, dilated fundoscopy, Goldmann applanation tonometry, and central corneal thickness measurement via ultrasonic pachymetry. They were then screened using an OCTA device (RTVue XR Avanti, version 2017.1.0.151; Optovue, Inc., Fremont, CA, USA) after pupillary dilatation in a dark room. All the OCTA measurements were recorded by the same individual, taking into account only images with a signal strength of *>*8. Poor-quality images with a signal strength of <8, presence of one or more blink artifacts, poor fixation resulting in motion or doubling artifacts, and segmentation errors were excluded. The scanning area captured in our study consisted of 4.5 x 4.5 mm sections centered on the ONH.

**Figure 1:**
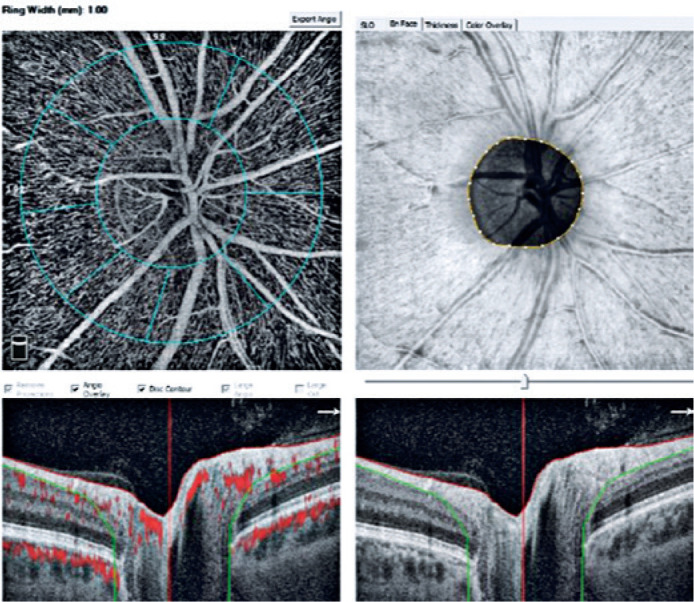
A radial peripapillary capillary map of an eye showing two optic nerve head centered concentric circles with diameters of 2 mm (inner) and 4 mm (outer ring width: 1 mm).

**Figure 2:**
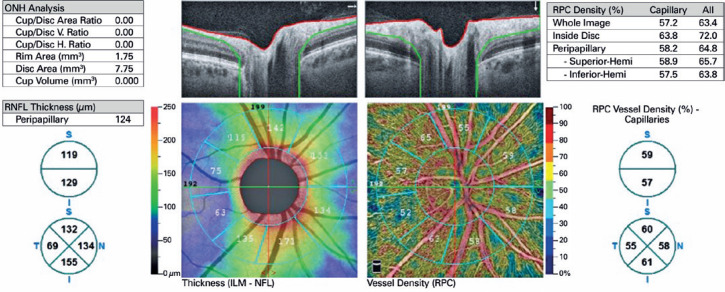
Optical coherence tomography angiography image of an eye with the radial peripapillary capillary density, retinal nerve fiber layer thickness values, and the optic nerve head parameters.

The device automatically attached two concentric circles, centered around the ONH, with diameters of 2 mm (inner) and 4 mm (outer) (ring width: 1 mm) (Figure 1). The RPCD was evaluated between these rings, inside the disk, peripapillary region, in 4 quadrants (superior, inferior, nasal, and temporal), and in 2 equal hemispheres (superior and inferior) using the OCTA density assessment tool (Figure 2). Large retinal vesselrelated flow signals were removed by the recent Angio DiscVue OCT software update (Phase 7). The segmentation was between the inner limiting membrane and the RNFL. The peripapillary RNFL thickness (RNFLT) was also determined peripapillary region, in the 4 quadrants, and in the 2 equal hemispheres, as with the RPCD, via the ONH analysis of the Angio DiscVue. The C/D area ratio, C/D vertical ratio, C/D horizontal ratio, rim area (mm^2^), disk area (mm^2^), and cup volume (mm^3^) were also measured using the ONH analysis of the Angio DiscVue (Figure 2).

### Statistical analysis

The right eye of each patient was evaluated in this study. The statistical analysis was performed using the Statistical Package for the Social Sciences (SPSS) software for Windows (version 17.0; SPSS Inc., Chicago, IL, USA). The Kolmogorov-Smirnov test was used to determine whether the values had normal distributions. The independent samples *t*-test was used to compare the parameters of the *H. pylori*-positive and -negative groups. The significance level for all the tests was set at p<0.05.

## RESULTS

The demographic and clinical characteristics of all of the patients are shown in table 1. The mean age was 42.57 ± 10.23 years for Group 1 and 41.29 ± 10.20 years for Group 2 (p=0.56). There were 26 females and 26 males in Group 1, and 24 females and 14 males in Group 2 (p=0.19). The groups were similar in terms of the BCVA, IOP, and central corneal thickness values (p=0.29, p=0.15, and p=0.53, respectively) (Table 1).

**Table 1:** Demographical and clinical characteristics of patients with or without *Helicobacter pylori* infection

Parameter	H. Pylori (positive) (Group 1)	H. Pylori (negative) (Group 2)	p-value
Patient (eye number)	52 (52 eyes)	38 (38 eyes)	
Age, mean ± SD year	42.57 ± 10.23	41.29 ± 10.20	0.56†
(range)	(21-60)	(26-60)	
Gender: Female/Male	26/26	24/14	0.19††
BCVA, mean ± SD	0.99 ± 0.01	0.99 ± 0.02	0.29+
1OP, mean ± SD (mmHg)	14.13 ± 2.66	13.36 ± 1.99	0.15^†^
Pachymeter, mean ± SD (µm)	534.33 ± 19.74	537.11 ± 21.55	0.53+

BCVA= best-corrected visual acuity; *H. pylori*= *Helicobacter pylori* pressure; SD= standard deviation. *p*-value

† = independent samples t-test, *p*-value

†† = Chi-square test.

Table 2 shows the ONH parameters of all the patients. There were no significant differences between the groups with regard to the C/D area, C/D vertical ratio, C/D horizontal ratio, rim area, disk area, and cup volume (p >0.05).

**Table 2:** The optic nerve head parameters of all patients

**Parameter**	H. Pylori (positive) (Group 1)	H. Pylori (negative) (Group 2)	**p-value**
C/D area	0.14 ±0.11	0.14 ±0.14	0.81†
C/D vertical	0.34 ± 0.21	0.29 ± 0.26	0.36+
C/D horizontal	0.30 ± 0.19	0.26 ±0.23	0.33†
Rim area (mm^2^)	1.74 ± 0.37	1.67 ± 0.37	0.37+
Disc area (mm^2^)	2.05 ± 0.40	1.96 ± 0.44	0.41†
Cup volume (mm^3^)	0.06 ± 0.06	0.05 ± 0.07	0.76†

C/D= Cup/Disc; *H. pylori: Helicobacter pylori*. The values are presented as mean ± standard deviation. *p*-value

† = independent samples t-test.

The RNFLT values are shown in table 3. The groups were similar in terms of the RNFLT in the superior and inferior quadrants, superior and inferior hemispheres, and peripapillary area (p >0.05 for all the values). However, the RNFLT values in the nasal and temporal quadrants were significantly lower in patients with *H. pylori* infections compared to those of patients without *H. pylori* infections (p=0.01 and p=0.02, respectively).

**Table 3:** Retinal nerve fiber layer thickness measurements of all patients

Parameter RNFLT (µm)	H. Pylori (positive) (Group 1)	H. Pylori (negative) (Group 2)	p-value
Peripapillary	113.66 ± 14.15	112.85 ± 11.29	0.77^†^
Superior hemisphere	114.19 ± 16.13	111.94 ± 13.12	0.49^†^
Inferior hemisphere	113.17 ± 14.67	114.00 ± 11.80	0.78^†^
Superior quadrant	133.53 ± 17.94	134.29 ± 22.29	0.86^†^
Inferior quadrant	143.23 ± 20.07	148.00 ± 16.49	0.24^†^
Nasal quadrant	98.44 ± 11.72	107.85 ± 22.50	0.01^†^
Temporal quadrant	71.48 ± 10.02	76.47 ± 10.72	0.02^†^

RNFLT= retinal nerve fiber layer thickness; *H. pylori*= *Helicobacter pylori.*

The values are presented as mean ± standard deviation. *p*-value^†^= independent samples t-test.

**Figure 3:**
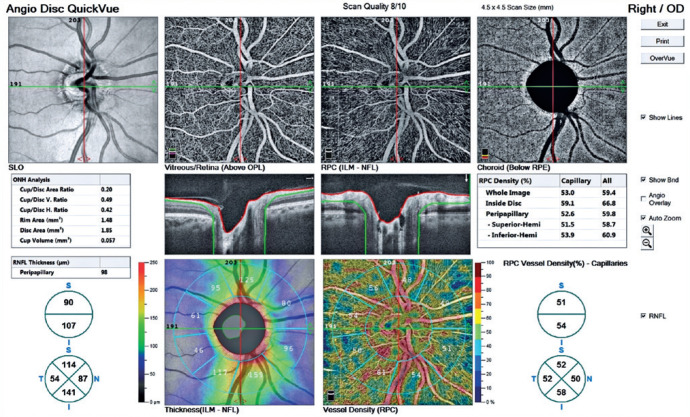
Optical coherence tomography angiography image of *H. pylori-*positive eye.

**Figure 4:**
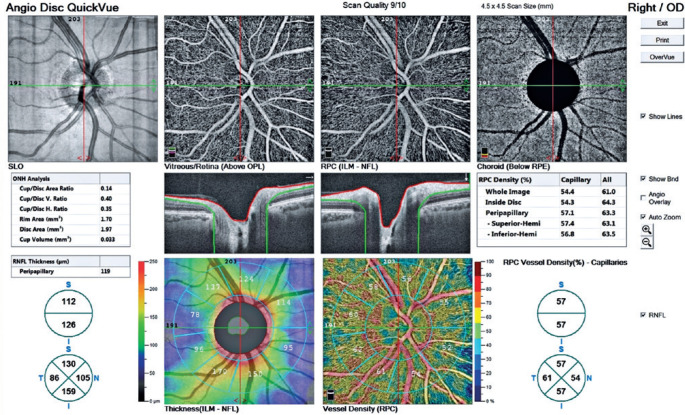
Optical coherence tomography angiography image of *H. pylori-*negative eye.

Table 4 summarizes the RPCD values of the 2 groups. The RPCD values of the whole image, inside disk, peripapillary region, nasal quadrant, and temporal quadrant were similar between the groups (p >0.05). However, the superior and inferior hemispheres, and superior and inferior quadrants showed significantly lower RPCD values in the *H. pylori*-positive patients than in the *H. pylori*-negative patients (p=0.03, p=0.03, p=0.02, and p=0.01, respectively). The OCTA images of patients with and without *H. pylori* infection are shown in figures 3 and 4, respectively.

**Table 4:** Radial peripapillary capillary vessel density measurements of all patients

Parameter RPCD (%)	H. Pylori (positive) (Group 1)	H. Pylori (negative) (Group 2)	p-value
Whole image	50.60 ± 2.73	51.40 ± 2.73	0.18^†^
Inside disc	51.24 ± 5.00	52.45 ± 4.16	0.24^†^
Peripapillary	52.87 ± 3.24	54.18 ± 3.31	0.06^†^
Superior hemisphere	52.22 ± 3.33	54.19 ± 3.40	0.03^†^
Inferior hemisphere	52.48 ± 3.58	54.17 ± 3.48	0.03^†^
Superior quadrant	52.03 ± 4.97	54.35 ±4.42	0.02^†^
Inferior quadrant	53.23 ± 4.21	55.55 ± 4.65	0.01^†^
Nasal quadrant	54.83 ± 5.33	55.76 ± 6.01	0.44^†^
Temporal quadrant	51.55 ± 5.86	52.52 ± 6.38	0.46^†^

RPCD= radyal peripapillary capillary density; *H. pylori: Helicobacter pylori*.

The values are presented as mean ± standard deviation. *p*-value

† = independent samples t-test.

Table 5 shows the correlation between the RPCD values and RNFLT values. There were significant positive correlations between these 2 parameters in the peripapillary region, all the 4 quadrants, and both the hemispheres (p<0.05).

**Table 5:** The correlations between radial peripapillary capillary density and retinal nerve fiber layer thickness of patients with *Helicobacter pylori* infection

Parameter	Peripapillary RPCD	Superior-hemi RPCD	Inferior-hemi RPCD	Superior quadrant RPCD	Inferior quadrant RPCD	Nasal quadrant RPCD	Temporal quadrant RPCD
Peripapillary RNFLT	r=0.452 *p*<0.01	r=0.416 *p*<0.01	r=0.438 *p*<0.01	r=0.382 *p*<0.001	r=0.412 *p*<0.001	r=0.992 *p*<0.01	r=0.316 *p*=0.002
Superior-hemi RNFLT	r=0.371 *p*=0.001	r=0.371 *p*=0.001	r=0.328 *p*=0.002	r=0.286 *p*=0.006	r=0.287 *p*=0.006	r=0.704 *p*=0.001	r=0.241 *p*=0.022
Inferior-hemi RNFLT	r=0.448 *p*<0.01	r=0.370 *p*<0.001	r=0.478 *p*<0.01	r=0.414 *p*<0.001	r=0.474 *p*<0.001	r=0.620 *p*<0.001	r=0.33 *p*=0.001
Superior quadrant RNFLT	r=0.325 *p*=0.002	r=0.351 *p*=0.001	r=0.260 *p*=0.013	r=0.316 *p*=0.002	r=0.247 *p*=0.019	r=0.649 *p*=0.001	r=0.237 *p*=0.019
Inferior quadrant RNFLT	r=0.412 *p*<0.01	r=0.330 *p*=0.002	r=0.451 *p*<0.01	r=0.427 *p*<0.001	r=0.453 *p*<0.001	r=0.750 *p*=0.002	r=0.244 *p*=0.020
Nasal quadrant RNFLT	r=0.264 *p*=0.012	r=0.237 *p*=0.024	r=0.262 *p*=0.013	r=0.255 *p*=0.014	r=0.212 *p*=0.045	r=0.484 *p*=0.002	r=0.288 *p*=0.006
Temporal quadrant RNFLT	r=0.390 *p*<0.01	r=0.347 *p*=0.001	r=0.390 *p*<0.01	r=0.309 *p*=0.003	r=0.432 *p*<0.001	r=0.397 *p*=0.001	r=0.241 *p*=0.022

RPCD= radial peripapillary capillary density; RNFLT= retinal nerve fiber layer thickness; Hemi= hemisphere.

r= Pearson correlation coefficient; the values of *p*<0.05 are significant.

## DISCUSSION

Glaucoma is a progressive optic neuropathy that causes irreversible blindness. Although an increased IOP is the most important risk factor, it is not the only precursor for this neurodegenerative condition^(22)^. Glaucoma progression continues despite having a normal IOP, as in patients with NTG, indicating that vascular optic neuropathic conditions such as an inadequate blood supply to the retinal ganglion cells and optic nerve fibers should also be taken into account in the etiopathogenesis of glaucoma^(14)^.

*H. pylori* infection has recently been accused of being a glaucoma etiology. Although the relationship between glaucoma and *H. pylori* is still a controversial issue, there is increasing evidence that *H. pylori* may be one of the causes of glaucoma. Studies on the relationship between glaucoma and *H. pylori* began in the early 2000s^(12,23,24)^, when a higher incidence of *H. pylori* in patients with PxG than in the control group was reported^(23)^. This link was supported by the detection of positive IgG antibodies of *H. pylori* in the anterior chamber of the eyes of patients with POAG and PxG when compared to the control group^(12)^. It was further confirmed when it was demonstrated that the eradication of *H. pylori* slowed down the progression of glaucoma in chronic, open-angle glaucoma patients^(24)^. Another researcher who noted that the incidence of *H. pylori* in patients with NTG was higher than in the normal population, pointed out that *H. pylori* may be an important factor in the etiopathogenesis of glaucoma^(14)^.

*H. pylori* is one of the most common chronic bacterial infections worldwide^(25)^. Glaucoma is an optic neuropathy that depends on several concomitant factors, making it difficult to clearly identify the factor that triggers it. This adds to the confusion in understanding the relationship, if any, between *H. pylori* and glaucoma. We compared the RNFLT in *H. pylori-*positive and -negative patients without known glaucoma and/or glaucomatous optic nerves in our previous study and found a significantly thinner temporal quadrant RNFLT in the *H. pylori*-positive patients^(3)^.

Vascular insufficiency in the ONH, which may be associated with changes in the endothelium-dependent vascular regulation and impaired ocular blood flow resulting from blood hyperviscosity^(26)^, is an important factor in glaucoma etiopathogenesis. It is believed that *H. pylori* may cause glaucoma through similar mechanisms. Long-term *H. pylori* infections lead to occlusive arterial diseases, such as atherosclerosis. Endothelial dysfunction induced by vacuolating cytotoxin A secreted from *H. pylori,* molecular mimicry by the autoimmune response, enhanced systemic inflammation, oxidative stress, and platelet aggregation are all potential mechanisms of atherosclerosis induced by an *H. pylori* infection. An increase in the inflammatory cytokines affecting the microvascular vasomotor mechanisms, such as interleukin (IL)-1, IL-6, tumor necrosis factor-alpha, elevated C-reactive protein in blood, intercellular adhesion molecule-1, and high homocysteine levels have been shown to confirm its role in endothelial dysfunction and atherosclerosis^(23)^.

OCTA, a novel advancement of OCT, allows the visualization and objective evaluation of the RPC network, which is thought to feed the RNFL in the peripapillary field. Ischemia in the ONH due to a decrease in the RPCD is considered to play a role in the pathogenesis of glaucoma^(26)^. Some previous studies have demonstrated a reduction in the RPCD in patients with glaucoma, including NTG^(17-19)^. A decrease in the RPCD due to decreased blood flow occurs first, causing ischemia of the nerve fibers and RNFL thinning^(27,28)^. A strong association between RPCD reduction and thinning of the RNFL has been previously established by many studies. In our study, although the RPCDs in both the superior and inferior hemispheres and quadrants were found to be significantly lower in patients with *H. pylori* infections compared to the *H. pylori*-negative patients, there was no significant corresponding thinning of the RNFLT noted in those areas. However, the RNFLT in the nasal and temporal quadrants of patients with *H. pylori* was found to be lesser than that of patients without *H. pylori,* without any corresponding changes in the RPCDs in the same quadrants. Despite the strong relationship between the RPCD and RNFLT, OCTA images have not been able to determine exactly which one occurred first-changes in the ocular blood flow or optic nerve injury^(29)^ RNFL thinning may be secondary to a reduction in the RPCD, as mentioned above, or it may be secondary to the loss of the surrounding RNFL from a primarily neuropathic process, without any changes in the RPCD via a neurovascular coupling mechanism^(30)^. In addition, we found a significant positive correlation between the RPCD and RNFL in all the areas (p<0.05).

The limitations of our study were the short follow-up period and lack of an RPCD re-evaluation after *H. pylori* eradication. However, in our previous study, we did not detect any improvement in the RNFL thickness after *H. pylori* eradication^(3)^.

In conclusion, decreased RPCD may be detected in patients with *H. pylori* infection using OCTA. Further longitudinal prospective studies with larger patient groups are needed to determine if there is a relationship between decreased RPCD and glaucoma in *H. pylori-* positive patients.
